# Androgen receptor and nutrient signaling pathways coordinate increased amino acid transport in prostate cancer progression

**DOI:** 10.1186/1753-6561-6-S3-P23

**Published:** 2012-06-01

**Authors:** Qian Wang, Charles G Bailey, Cynthia Ng, Jessamy Tiffen, Annora Thoeng, Vineet Minnas, Melanie L Lehman, Stephen C Hendy, Grant Buchanan, Colleen C Nelson, John EJ Rasko, Jeff Holst

**Affiliations:** 1 Origins of Cancer Laboratory, Centenary Institute, Camperdown, Australia; 2 Gene & Stem Cell Therapy Program, Centenary Institute, Camperdown, Australia; 3 Sydney Medical School, University of Sydney, Australia; 4 Vancouver Prostate Centre, Department of Urologic Sciences, University of British Columbia, Vancouver, Canada; 5 Molecular Ageing Laboratory, University of Adelaide, Australia; 6 Australian Prostate Cancer Research Centre-Queensland, Queensland University of Technology, Australia; 7 Cell and Molecular Therapies, Royal Prince Alfred Hospital, Camperdown, Australia

## Background

Solid tumors including prostate cancer activate angiogenic signals to ensure an adequate blood supply. In parallel, amino acid transporters on the cell surface are also increased so as to provide nutrients for the higher metabolic and growth demands of cancers. We are studying the L-type amino acid transporters (LAT1 and LAT3) that mediate uptake of essential amino acids including leucine. Leucine has recently been shown to be critical for the activity of mTORC1, which regulates protein translation and cell growth. Therefore, increased amino acid transport in prostate cancer cells may drive the mTORC1 signaling pathway to promote unrestrained cellular proliferation.

## Materials and methods

We have used the androgen dependent (LNCaP) and androgen independent (PC-3) prostate cancer cell lines to test the role of amino acid transport in cancer. We have used both amino acid uptake inhibitors (BCH) and shRNA knockdown to test the effects on *in vitro* cell growth, cell cycle and signaling pathway analysis as well as *in vivo* bioluminescent tumor growth assays and clinical data correlations with experimental data from primary human cancers.

## Results

Our results have demonstrated that prostate cancer cells coordinate the expression of LAT1 and LAT3, thereby increasing leucine uptake to promote mTORC1 signaling and cell growth [1]. We show that inhibition of LAT function leads to decreased cell growth and mTORC1 signaling in prostate cancer cells. These cells maintain amino acid influx via androgen receptor regulation of LAT3 expression, and ATF4 regulation of LAT1 expression after amino acid deprivation. These responses are intact in primary prostate cancer, as indicated by high levels of LAT3 in primary disease, and an increase in LAT1 following hormone ablation and in metastatic lesions. This dynamic regulation of transporter expression is also seen in LNCaP tumor xenograft models, whereby castration decreases LAT3 expression and increases LAT1 expression. Furthermore, shRNA knockdown of either LAT1 or LAT3 significantly decreased tumor growth *in vivo.*

## Conclusions

These data show that prostate cancer cells respond to the demand for increased amino acids through an integrated pathway, leading to increased amino acid transporter expression and cell growth. Furthermore, LAT3 and LAT1 may provide novel therapeutic targets in early and late stage prostate cancer respectively.
